# An Experimental Comparison of IoT-Based and Traditional Irrigation Scheduling on a Flood-Irrigated Subtropical Lemon Farm

**DOI:** 10.3390/s21124175

**Published:** 2021-06-17

**Authors:** Huma Zia, Ahsan Rehman, Nick R. Harris, Sundus Fatima, Muhammad Khurram

**Affiliations:** 1 College of Engineering, Abu Dhabi University, Zayed City, Abu Dhabi P.O. Box 59911, United Arab Emirates; 2 Smart City Lab, NCAI (National Center of Artificial Intelligence), NED University of Engineering and Technology, Karachi 75270, Pakistan; ahsanrehman@neduet.edu.pk (A.R.); sundussfatima@neduet.edu.pk (S.F.); mkhurrum@neduet.edu.pk (M.K.); 3 Electronics and Computer Science, University of Southampton, Southampton SO17 1BJ, UK; nrh@ecs.soton.ac.uk

**Keywords:** smart irrigation, decision support system, Internet of things, Penman–Monteith equation, crop coefficient

## Abstract

Over recent years, the demand for supplies of freshwater is escalating with the increasing food demand of a fast-growing population. The agriculture sector of Pakistan contributes to 26% of its GDP and employs 43% of the entire labor force. However, the currently used traditional farming methods such as flood irrigation and rotating water allocation system (Warabandi) results in excess and untimely water usage, as well as low crop yield. Internet of things (IoT) solutions based on real-time farm sensor data and intelligent decision support systems have led to many smart farming solutions, thus improving water utilization. The objective of this study was to compare and optimize water usage in a 2-acre lemon farm test site in Gadap, Karachi, for a 9-month duration, by deploying an indigenously developed IoT device and an agriculture-based decision support system (DSS). The sensor data are wirelessly collected over the cloud and a mobile application, as well as a web-based information visualization, and a DSS system makes irrigation recommendations. The DSS system is based on weather data (temperature and humidity), real time in situ sensor data from the IoT device deployed in the farm, and crop data (Kc and crop type). These data are supplied to the Penman–Monteith and crop coefficient model to make recommendations for irrigation schedules in the test site. The results show impressive water savings (~50%) combined with increased yield (35%) when compared with water usage and crop yields in a neighboring 2-acre lemon farm where traditional irrigation scheduling was employed and where harsh conditions sometimes resulted in temperatures in excess of 50 °C.

## 1. Introduction

Agriculture is the backbone of Pakistan’s economy, which contributes 26% of the country’s GDP and employs 43% of the total labor force [1]. The support of agriculture currently uses 60% of the total freshwater drawings [2]; 90% of land cultivation is achieved by irrigation using river water, and this contributes to over 80% of agricultural yield. However, the average rainfall is less than 240 mm a year [3], and it is estimated that the country may run dry by 2025 [4]. The climatic changes and high population growth (1.57% per year) in Pakistan require more water resources, not only for daily usage but also for agriculture [5]. However, the majority of farmers use outdated irrigation methods, such as flood irrigation, and rely on estimations for decision making in agricultural practices which typically result in excess water usage [6]. Additionally, a fixed rotational water allocation system, known as Warabandi, is used in the region for supplying irrigation water to farmers as per a preplanned schedule [7]. In this method, pre-allocated fixed volumes of irrigation water (estimated according to the crop) is provided into canals leading up to the fields, on a given day [8]. This method is widely used in Pakistan, India, Bangladesh, and some of the Mediterranean region [8,9]. This practice, although providing equitable water supply for all farmers in the region (which, otherwise, could result in conflicts over water usage) does not incorporate the actual irrigation requirements as per the field parameters on a given day. It provides access to water, at set times, but not all the time. This can result in untimely and excessive irrigation of the fields. Excess irrigation not only depletes already scarce freshwater resources, but it also affects the crop quality. The additional water in the soil reduces the necessary space required for plant respiration, which consequently hinders plant growth [10]. Excess water in the soil can also lead to salinity problems, which affects the fertility of the soil, thus reducing the available arable land [11]. Irrigation is highly dependent on three major factors—crop type, weather, and soil—that significantly affect the irrigation scheduling criteria [12]. Crop type and soil determines the volume of irrigation and fertilizers, whereas weather, soil moisture, humidity, and temperature govern the schedule of irrigation. Ultimately, the irrigation water requirements for crops change dynamically each year [13]. Thus, it is necessary to implement a scheduled irrigation policy for crops on the basis of these real time parameters for smart water management and high-quality crop yields [14]. Data-driven irrigation scheduling systems comprise two integral parts—dynamic models (based on weather, crop, and soil data) and an agriculture decision support system (DSS). The agriculture DSS analyzes the data, makes decisions based on real-time inputs of the soil, and approximates the time for the next irrigation. A DSS that uses machine learning for irrigation water management was presented in [15], and a study for decision systems and its use in water management was presented by Guariso et al. in [16]. The real-time calculation of the soil–water balance is essential for real-time irrigation scheduling. Real-time computations require the prediction of daily reference evapotranspiration (ET_o_), which is the basis for estimating crop evapotranspiration (ET_c_) and for computing crop irrigation requirements [17]. The FAO Penman–Monteith (FAO-PM) method is recommended as the standard ET_o_ method [18] and it has long been accepted worldwide as a good ET_o_ estimator when compared with other methods [19,20,21]. Using ET_0_, the daily soil water deficit is then calculated on the basis of the soil water balance equation, which is a widely used irrigation scheduling method [22]. With the advent of the Internet of things (IoT), the availability of real-time weather and in situ soil data has revolutionized decision making for farmers in the agriculture sector. The estimations made by the IoT-based agriculture DSS are more accurate than human estimation as they are based on an accurate analysis of variable real-time data [23]. IoT-based and wireless sensor network (WSN)-based monitoring and decision making for irrigation systems have accurately predicted irrigation scheduling in the past using measured data, thereby increasing water productivity [24,25,26,27,28,29,30,31,32,33,34].

To date, various applications have explored the implementation of IoT-based DSS for scheduling irrigation in farms. Some studies used the soil water-based equation, while others used the soil moisture threshold method [17]. In [29], a system was developed with a cloud-based framework for irrigation in an urban setting for a multilevel plant pot. Each node contained a local decision-making system along with sensors and actuators. The local nodes communicated the data to the cloud through a central node, where data could be analyzed. The experiment, though a small-scale testbed, showed reduced water consumption. Machine learning was employed in another 3-week study [24] for an IoT-based irrigation management system on a small test bed. The system included embedded microcontroller base boards (Raspberry Pi and an Arduino) to recommend the irrigation requirements of a field using the predicted soil moisture value. The prediction algorithm used ground parameters such as soil moisture, soil temperature, and environmental conditions along with the weather forecast data from the Internet. The study demonstrated predictions of good accuracy (*R*^2^ = 96%) but did not analyze the water savings. The device was also not robust enough to be deployed in a harsh and remote environment. The studies in [24,25,28] also proposed IoT-based irrigation scheduling systems and deployed them on a pilot scale. The systems were based on direct soil water measurement to utilize water optimally compared to traditional irrigation systems.

Another study [33] focused on smart irrigation scheduling for tunnel farming. The researchers used sensors to acquire light, temperature, humidity, and soil moisture readings which were calibrated to deploy fuzzy logic and generate results that determined whether and when the crop requires water. The readings were forwarded to the server where they could be accessed and viewed by an Android (a popular and standardized mobile operating system from Google) application or a web browser. This study was unique as it focused on tunnel farming, deployed fuzzy logic to make smart decisions for scheduling, and saved energy consumption by controlling active times of sensors. A DSS for automatic irrigation scheduling based on machine learning methods was analyzed in a study in Spain [35]. The study was aimed toward testing different machine learning algorithms, such as linear regression, support vector machine, and random forest regression, for predicting irrigation values. The models were trained on agronomical data acquired from nine citrus orchards of 5–6 ha area each. For that, nodes developed by a smart solutions company were used. Weather data such as temperature, humidity, radiation, wind speed, rainfall, and vapor pressure were provided by a public research institute in Spain, which deployed 49 climate stations covering the region under study. The study showed weekly average error below 10% for the predictions in comparison to the values recommended by the agronomist. Another research study for lemon farming was conducted using an IoT device, in which soil moisture threshold values were used to trigger irrigation. The system included a hardware node based on a microcontroller unit, DHT22 sensor (temperature and humidity), and a soil moisture sensor along with a mobile application and a web portal. According to threshold values of soil moisture, the farmer can decide to irrigate the field. The study was actually aimed at finding the relationship between humidity and temperature on crop yield on the basis of season-long collected data, for which a linear regression model was used. It was determined that the suitable temperature and humidity for high productivity of homegrown lemons was 29–32 °C and 72–81%, respectively. Most of the earlier studies were small-scale pilot deployments and were not able to evaluate the impact of smart irrigation on total water savings and crop yields over a season. Furthermore, none of the studies were based on flood irrigation, which offers its own limitations. Moreover, Pakistan uses a traditional rotational water allocation method, Warabandi, which puts limitations on water scheduling and needs to be considered for the DSS. Some studies used public weather data, while others used weather data to predict soil moisture to schedule irrigation. 

There are various commercial IoT modules available for irrigation scheduling such as CropX sensor [36], Teralytic sensor [37], Mark2 sensor [38], and Libelium Kit [39]. The cost of these systems ranges from 1000 to 5000 USD. CropX measures soil moisture, soil temperature, and electrical conductivity at multiple depths of soil, as well as weather conditions [36]. It has its own communication modules and works as a standalone component. It costs about 600 USD and has shown water savings of up to 30%. The Teralytic sensor probe along with installation kit and gateway costs up to 4300 USD [37]. It measures soil NPK (nitrate, phosphates, potassium) levels, soil pH, soil moisture, soil temperature, and weather parameters. The sensor probe cannot work independently and requires the associated hub for data transfer. No published results could be found as per the authors’ best knowledge to demonstrate water savings using this device. The Mark2 sensor has similar features to the above modules and costs about 1600 USD [38]. The Libelium agriculture kit measures soil moisture, temperature, humidity, leaf wetness, and atmospheric pressure [39]. It also comes with a data visualization software. This kit costs 5000 USD, which includes nodes and a hub. The available tools and systems discussed above are not cost-effective, which increases the dependency of the users on imported solutions, which makes maintenance challenging. Hence, there is a requirement for a low-cost indigenously developed IoT device to address the specific regional requirements.

The proposed study represents the country’s first commercial smart irrigation system. Due to indigenous development, the system is likely to be maintained at lower cost against the imported solutions which are expensive to maintain. Such a system must utilize in situ soil moisture data, as well as real-time weather data, for estimating crop water requirements, and this is what we report on in this paper.

In this paper, we present the results of a 9-month long study on a 2-acre lemon farm, where the indigenously developed IoT device was deployed and monitored. The field was compared with a similar 2-acre lemon farm, where traditional farmer-controlled irrigation scheduling was used. Lemons, unlike rice and sugarcane, are not a water-intensive plant; hence, this offers significant scope for achieving water saving over traditional lemon farming techniques in the region. The real-time field and weather data were collected through a self-designed sensor node. An information visualization feature and DSS system was developed for both a mobile application and a web portal for the ease of the farmers to keep track of their crops and their water requirements. The novel agriculture DSS algorithm uses collected data to calculate daily grass reference evapotranspiration (ET_0_), crop coefficients (K_c_), and daily crop evapotranspiration ET_c_. The algorithm calculates soil water depletion and total water availability and recommends an irrigation schedule to the farmers, keeping in view the Warabandi system. Our study is novel in the sense that no other research in the region has targeted complete design, implementation, deployment, and evaluation of a self-designed low-cost IoT system based DSS solution (under 100 USD) for irrigation scheduling and crop yield monitoring for an entire season. Furthermore, no study has focused on the Warabandi system of irrigation. The authors have extensive research experience with regard to smart irrigation using IoT solutions and environmental modeling for farm water management [30,31,40]. The developed product was launched in a start-up company as a service and is being adopted by many farmers in the country for crop monitoring and irrigation scheduling [41].

## 2. Materials and Methods

**Figure 1:**
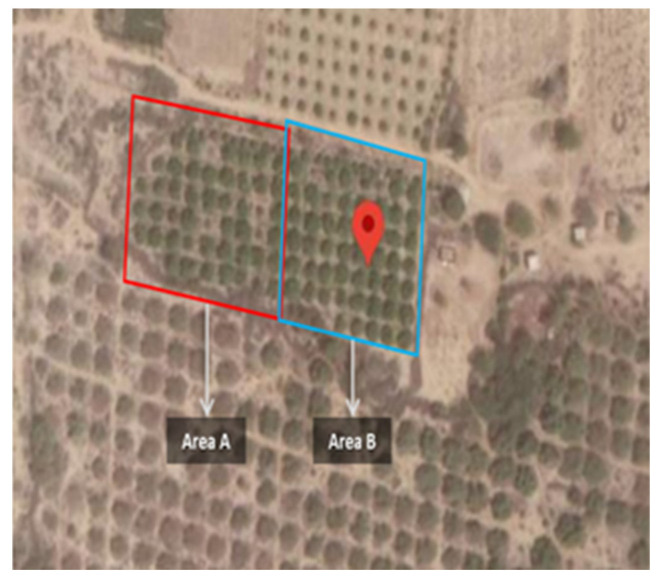
Area A for farmer estimations (2 acres); Area B for agriculture DSS recommendations (2 acres).

Our study highlights the difference between conventional irrigation and agriculture DSS-based irrigation. The study was conducted on a 4-acre farm in Gadap, Karachi, from February 2018 to October 2018 (9 months) on a lemon orchard. In total, 4 acres of land was used for the trial: 2 acres reserved for farmer-controlled irrigation (Area A) and 2 acres reserved for agriculture DSS-controlled irrigation (Area B), as shown in Figure 1. Flood irrigation was used in both the areas. Area A was irrigated as a function of the traditional estimations made by the farmers for irrigation time and volume, whereas Area B was irrigated using the recommendations made by the agriculture DSS as a function of real-time information provided by the deployed IoT device.

### 2.1. Implementation Detail

**Figure 2:**
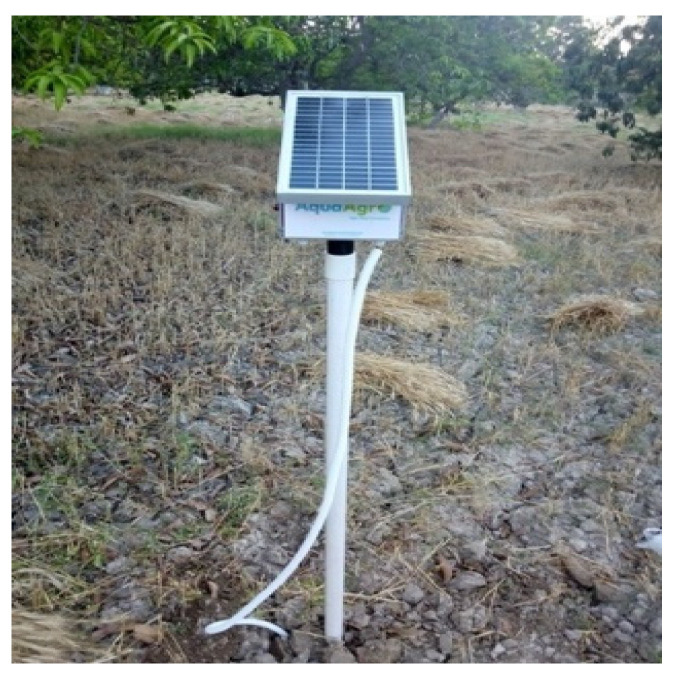
IoT device.

The bespoke IoT device (as shown in Figure 2), developed by the authors, was used in Area B to monitor soil and weather data in real time. The application uses third-party wind data (m/s) from online sources [42]. A DHT22 sensor [43] is embedded in the IoT device to collect temperature and humidity, and the frequency-domain reflectometry (FDR) soil moisture sensor [44] measures the soil moisture at 30 cm depth. The details of the IoT device are covered in the next section.

Before the final deployment at the beginning of the season in February, the system was developed and tested at the study site for about 4 months. Information related to soil, crop, and irrigation system was gathered with the help of expert farmers and incorporated in the DSS algorithm (discussed in the next section). The IoT device is enclosed in a hardware box to be securely deployed on the site. During initial testing, two devices were deployed in the 2-acre field to provide spatial resolution for data. However, due to no variability in soil parameters and weather conditions, as well as a leveled land for the test site and crop, observed parameters by the sensors did not vary. Hence, it was decided to only use a single a device for monitoring. The final results in terms of irrigation savings and yield increase validated our decision. Therefore, scalability is not applicable in our case; more sensors would only be needed if soil and weather parameters are likely to change on the given site. The cost of a single IoT device is 65 USD for the farmers to purchase. The total cost of a single device, as well as its deployment, testing, and maintenance, for an entire season comes to about 170 USD. This includes the cloud service charges and access to the mobile application, web portal, and SMS service.

Once the system is deployed, the collected data from the sensors are sent to an online server by the device, where the agriculture DSS uses these data to calculate water requirements. For irrigation scheduling in DSS, the widely used method of evapotranspiration/water balance (ET-WB) is applied, in which daily crop evapotranspiration (ET_c_) is estimated according to the methods outlined by Food and Agriculture Organization (FAO) [22]. Daily soil water deficit is calculated using the ET-WB equation to schedule irrigation events when the total water depletion exceeds the readily available water. The FAO-56 Penman–Monteith equation is used to calculate daily grass reference evapotranspiration (ET_0_). ET_c_ is estimated using FAO-56 Dual Crop Coefficient procedures [18]. All this information is made available to the farmer using a mobile application and a web portal. These values are then used by the farmer to schedule irrigation for Area B. Where mobile service is not available, farmers are informed using an SMS message about irrigation scheduling and field status. The farmers make use of the existing infrastructure for irrigating their field. After the 9-month study, the research was able to record and compare the total farm yield, yield per tree, revenue, and total water usage of both the farm areas, as discussed in Section 3.

Below, we discuss the design architecture including the IoT device and DSS algorithm, irrigation calculation methods, and the output parameters.

### 2.2. Design Architecture

**Figure 3:**
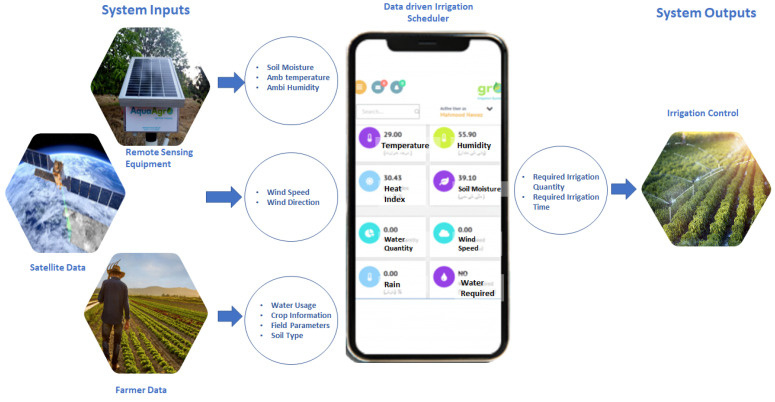
Overall architecture of agriculture-based DSS.

The comprehensive framework of the system, shown in Figure 3, illustrates the system input coming through the deployed IoT device in the farm (soil moisture, temperature, humidity), satellite data (wind speed and direction), and predefined data (crop type, soil type, location, plantation date).

The collected data are presented to the DSS using an underlying intelligent algorithm, which recommends the irrigation scheduling for the farm. The figure further illustrates the mobile application interface presented to the farmers to analyze the farm parameters and to get feedback on the irrigation requirement. We now discuss the two basic sections of the architecture—the IoT device and the DSS algorithm.

#### 2.2.1. IoT Device

**Figure 4:**
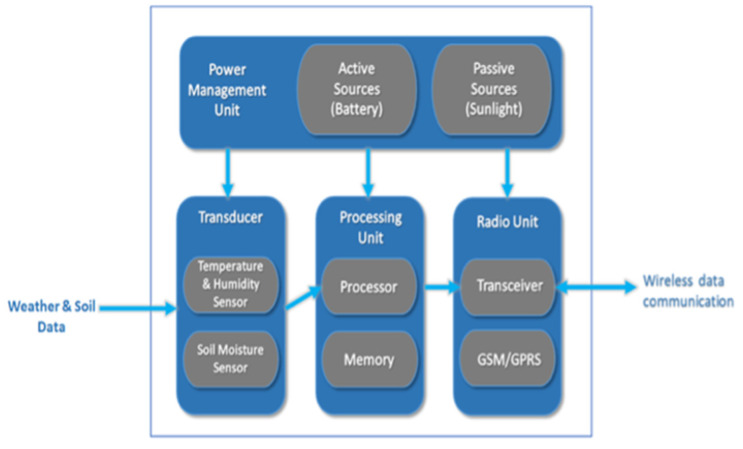
IoT device architecture.

The IoT node incorporates a power source, microcontroller, sensors, and RF transceiver for this application, as shown in Figure 4. Various sensors are used. The DHT 22 sensor and soil moisture sensor are used to measure the temperature and humidity of the environment, and the water content of the soil, respectively. All the sensors are interfaced with high-performance, low-power AVR series microcontrollers ATmega8/ATmega328. The pins of the controller are interfaced with sensors and the RF transceiver, through the serial port. The GSM/GPRS module is used for communication purposes and to send the recorded data to the cloud. A separate board for providing the sensors power was created. A 9 V lithium battery is used to provide the power. The battery capacity is 2000 mAh but the primary supply is from an 18 W solar cell. The battery allows a lifetime of 2 days without charging. It is regulated by a 78H05K regulator to maintain the voltage at 5 V for the microcontroller, which then further lowers the voltage to 3.3 V for the RF transceiver.

**Figure 5:**
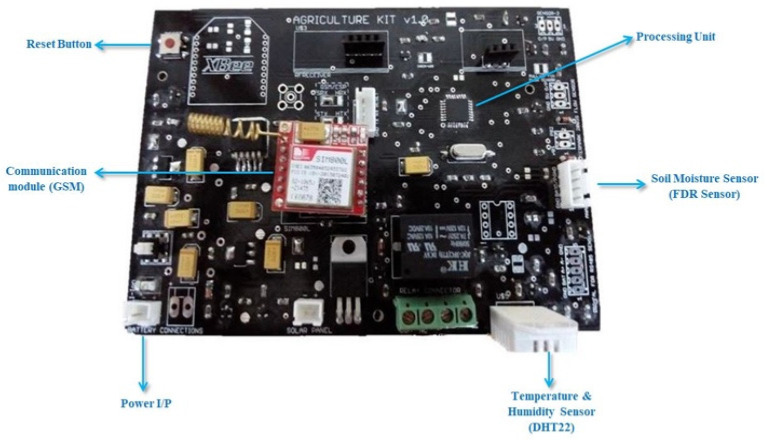
IoT PCB.

The device was placed in a plastic box under the solar panel for protection from the harsh weather conditions in a farm, which, in previous cases, affected the circuitry and casing of the device. However, the sensors were able to perform under the harsh weather conditions as the temperature peaked at 52 °C in the summer. The custom PCB of the IoT device is shown in Figure 5. The readings of temperature, soil moisture, and humidity were recorded by the sensors once per hour in a day and the data were sent to a cloud analytics platform service called ThingSpeak via the GSM module (SIM800 L). ThingSpeak is a cloud platform that aggregates and visualizes the data sent from devices (or sensors) [45]. This device can be used on any crop without any changes in the structure as the device is used for data acquisition. The collected data are then fed into the online DSS that combines the soil and weather data with crop data, which ultimately results in estimating the water requirements of the crop. Multiple devices can be installed on different crops at the same time without any change in the hardware design to collect the soil and weather data. The backend software deployed on an online server represents the crop’s predefined data for each device so that the system can accommodate all the crops if their predefined data are available.

#### 2.2.2. DSS Algorithm

**Figure 6:**
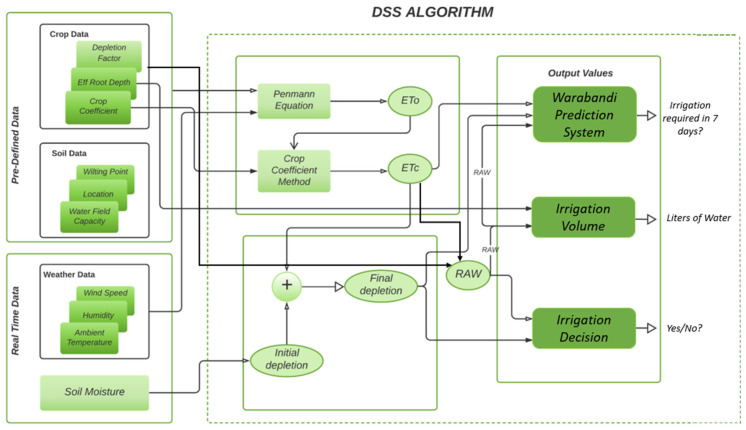
DSS algorithm.

The DSS algorithm determines if irrigation is required by the farmer or not, and how much irrigation volume is needed. The overall flow of the DSS algorithm is illustrated in Figure 6. The agriculture DSS functions on two types of data: real-time and predefined. The sensors present in the IoT device measure real-time ambient temperature, humidity, and soil moisture data. The values from the sensor are dynamic as they change every hour. These hourly updated values from the sensors are necessary for the agriculture DSS to make accurate decisions. Furthermore, the DSS uses third-party monthly average wind data (m/s) from an online source for the Gadap region [42]. The wind parameters are required to calculate ET_0_ but do not need to be measured on the node as they are available from online sources.

Some predefined static data are used in the system, such as crop data, soil data, and location data. Crop data include crop coefficient, effective root depth, and planting date. Soil data include soil type, soil water capacity, and wilting point. Location data include longitude and latitude of the field. These values are preset prior to installing the device in the field and are essential for the agriculture DSS to optimally schedule irrigation. 

To assess if the irrigation is needed on a given day, there is a fundamental condition recommended by Food and Agriculture Organization (FAO) [22] which is checked in the DSS algorithm daily: if D_r,i_ ≥ RAW, irrigation is needed, where D_r,I_ is the root zone depletion at the end of the i-th day (mm) and RAW is the readily available water in the root zone (mm).

This is the fundamental equation for the decision support system to daily calculate the irrigation decision for a particular area and crop. D_r,i_ is referred to as final depletion in this research. RAW represents the amount of water that must be maintained above final depletion value to avoid crop water stress and ensure proper crop growth. RAW is calculated using ET_c_ and predefined crop data. Every day, the two most critical values (depletion before evapotranspiration (initial depletion) and depletion after evapotranspiration (final depletion)) are calculated using field water capacity, mean soil moisture, and effective root depth information. The depletion values increase each day due to crop evapotranspiration. Eventually, depletion rises above the value of RAW, which means that irrigation is needed. The crop suffers if depletion remains greater than RAW. For ET_c_ and ET_c_ calculations, the Penman–Monteith model and crop coefficient approach are used, respectively (discussed later in detail). As Figure 6 illustrates, ET_o_ is calculated using daily temperature, daily humidity, wind speed, and location information (latitude and latitude). Once the above irrigation condition is true, the value of RAW and the total area covered by all the lemon trees are used to determine the required irrigation volume. The algorithm determines the duration (in minutes) as per the given water flow for irrigating a specific area.

As mentioned earlier, the Warabandi system is a rotational water allocation mechanism through which a fixed amount of water is supplied to each farmer on a given day. Hence, the irrigation DSS must take into account the limitations offered by the system and recommend irrigation according to the farmer’s turn without affecting crop health. As a result, the DSS system informs farmers to irrigate crops when the requirement for water is nearest to their turn. For example, if the agriculture DSS calculates that a crop requires water on Monday, but the turn of the farmer is on Friday, then the agriculture DSS will tell the farmer in advance to irrigate the crop on the previous Friday. Irrigating the crop on the previous Friday, before Monday, would be necessary to prevent wilting beyond Monday. Currently, no other organization or individual has developed a similar algorithm. Thus, the prediction algorithm in the DSS is novel and unique.

### 2.3. Irrigation Calculation Methods

For controlled irrigation, it is necessary to determine the amount of water the crop loses during a period, as well as the amount the crop requires. Water requirement changes with every crop type and region; however, there are two means by which water loss occurs. The first is evaporation of the water from the soil. The second is transpiration from the plant. Therefore, it is important to calculate how evaporation and transpiration (or evapotranspiration) affect the plant. Evapotranspiration is a combination of both terms, and this research focuses on calculating evapotranspiration as it allows researchers to efficiently estimate the water requirement of crops.

#### 2.3.1. Penman–Monteith 

The Penman–Monteith equation [46] (Equation (1)) is an effective method for calculating reference crop evapotranspiration (ET_0_).

(1) $${\mathrm{ET}}_{0}=\frac{0.408\Delta \left(R_{n}-G\right)+\Upsilon \frac{900}{T\;+\;273}u_{2}\left(e_{s}-e_{a}\right)}{\Delta +\Upsilon \left(1+0.34u_{2}\right)},$$

where ET_0_ is the reference evapotranspiration (mm per day), R_n_ is the net radiation at the crop surface (MJ/m^2^ per day), G is the soil heat flux density (MH/m^2^ per day), T is the mean daily air temperature at 2 m height (°C), u_2_ is the wind speed at 2 m height (m/s), e_s_ is the saturation vapor pressure (kPa), e_a_ is the actual vapor pressure (kPa), e_s_ − e_a_ is the saturation vapor pressure deficit (kPa), Δ is the slope of vapor pressure curve (kPa per °C), and γ is the psychrometric constant (kPa per °C).

The sensor provides the ambient temperature (T), and soil moisture. The remaining values are determined as described below.

##### Net Radiation (R_n_)

R_n_ is the radiation at the surface that can increase the accuracy of estimation if available. The value of R_n_ is calculated from publicly available libraries that implement an estimation formula, namely, meteolib [47] and FAO [48]. Different parameters are taken into consideration such as maximum and minimum values of temperature, latitude, and longitude to determine the final value of R_n_. 

##### Soil Heat Flux (G) 

The amount of thermal energy that moves through an area of soil per unit time is called the soil heat flux [46]. The agriculture DSS calculates ET_o_ every 24 h, and the value of soil heat flux is so small that it is considered to be negligible. Therefore, soil heat flux is G ≈ 0.

##### Wind Speed (u_2_)

(2) $$u_{2}=u_{z}\frac{4.87}{\ln\left(67.8z-5.42\right)},$$

where z is the elevation above sea level (m). 

FAO recommends keeping the sensors at 10 m above the surface because wind speed is lowest at ground level and increases with height. Therefore, anemometers are placed at a standard height, i.e., 10 m in meteorology and 2 or 3 m in agrometeorology. In this case, the altitude must be 2 m in the equation for best outcomes, and the dataset was recorded with wind speed at 10 m. This applies to the third-party wind datasets that were used for the calculation [42].

##### Saturation Vapor Pressure (e_s_)

Saturation vapor pressure, required in Equation (1), is calculated as shown in Equation (3).

(3) $$e_{s}=\frac{e^{o}\left(T_{\max}\right)+e^{o}\left(T_{\min}\right)}{2},$$

where T is the temperature (°C) and e^0^ (T) is the saturation vapor pressure at the air temperature T (kPa).

(4) $$e^{0}\left(T\right)=0.6108\exp\left[\frac{17.27T}{T+237.3}\right].$$

Equation (4) shows nonlinearity in computing the desired outcomes. Therefore, mean saturation vapor pressure is calculated for a day, month, or week at the mean maximum and minimum temperatures for a specific period of time.

##### Actual Vapor Pressure (e_a_)

e_a_ is calculated as shown in Equation (5).

(5) $$e_{a}=\frac{e^{0}\left(T_{\max}\right)\frac{{\mathrm{RH}}_{\max}}{100}+e^{0}\left(T_{\min}\right)\frac{{\mathrm{RH}}_{\min}}{100}}{2},$$

where T is the temperature (°C).

##### Vapor Pressure Curve (Δ)

Δ is determined using Equation (6).

(6) $$\Delta =\frac{4098\left[0.618\exp\left(\frac{17.27T}{T+237.3}\right)\right]}{{\left(T+237.3\right)}^{2}},$$

where T is the temperature (°C).

##### Psychometric Constant (γ)

The psychrometric constant, γ, is defined by the value of atmospheric pressure as shown in Equation (7), which is calculated using Equation (8). γ is related to the evapotranspiration from leaves, the rate of which is dependent on atmospheric pressure.

(7) $$\gamma =0.665\times 10^{-3}P,$$

where P is the atmospheric pressure (mb), as given by Equation (8).

(8) $$P=101.3{\left(\frac{293-0.0065z}{293}\right)}^{5.26},$$

where z is the altitude above sea level (m).

#### 2.3.2. Crop Coefficient Approach

The evapotranspiration, ET_0_, calculated using Penman–Monteith equation is used to estimate the reference crop evapotranspiration, ET_c_. Each crop has a unique evapotranspiration; therefore, the Penman–Monteith equation assigns ET_0_ to each crop. ET_0_ depends on real-time and predefined data. It considers variables such as wind, humidity, altitude, and latitude. Therefore, it uses a diverse dataset that includes real-time and predefined data.

To calculate ET_c_, the crop coefficient approach is used as shown in Equation (9).

(9) $${\mathrm{ET}}_{c}=K_{c}{\;\mathrm{ET}}_{0},$$

where K_c_ refers to crop coefficient, which is specific to various crops and their growth stages. For lemon, these values range between 0.6 and 0.8 according to FAO for various crop stages, changing with time [18].

### 2.4. System Outputs

The agriculture DSS provides the farmer with multiple options with respect to the irrigation output variable, as well as the user application interface. The user application interface includes a mobile application and a web portal.

#### 2.4.1. Output Parameter Options for Irrigation

The agriculture DSS offers flexibility to farmers to use different output parameters for irrigation as per their requirement. Some farmers calculate irrigation output in terms of volume, such as gallons or liters. Other farmers use acre per inch irrigation calculations. Furthermore, some farmers cannot calculate the volume or acre per inch, and they use time as an output. Therefore, the agriculture DSS provides the option for various output parameters to the farmers as per their requirement. For example, if farmers use time as an output term, then the system will tell them to irrigate for a specific duration. The terms of output do not affect the actual requirement of the crop. For example, if a crop requires 5 L of water, then the system delivers 5 L of water, whether the output is in volume, time, or acre per inch. It adjusts the output according to the required output term; it calculates how much time or acre per inch is equal to 5 L of water, and then shows the correct output amount to the farmer. Therefore, the agriculture DSS can work on any farm in Pakistan, with varying irrigation output parameter requirements. 

#### 2.4.2. Mobile Application and Web Portal

**Figure 7:**
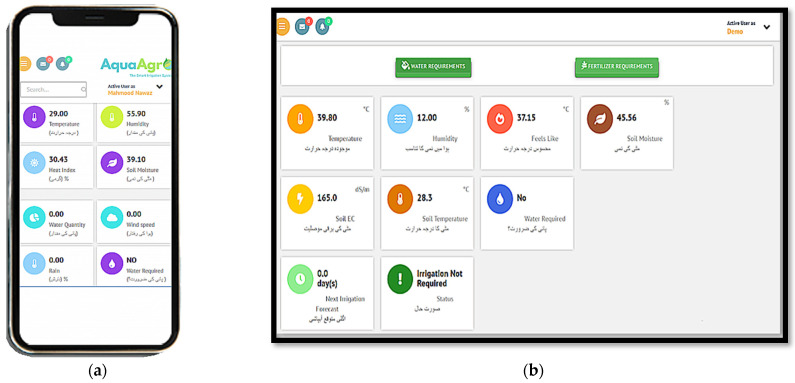
(**a**) Android application and (**b**) web portal for information visualization for the farmer.

An easy-to-navigate user interface is fundamental to promoting a technical farming solution for farmers in Pakistan. With the availability of mobile phones, information sharing has become more prevalent. Hence, for the agriculture DSS, a custom mobile application was developed to inform the farmers about the status of their crop using real-time data, as shown in Figure 7. The registered farmers can log into their account and access information related to the irrigation requirements of their farm. The application represents irrigation output parameters set as per the farmer’s preference. Different parameters such as temperature, humidity, soil moisture, soil temperature, and irrigation requirement are included on the main page of the application. The application alerts the farmer when irrigation is required at the farm. Farmers irrigate the crops according to irrigation output and schedule in the mobile application. In addition, an SMS alert service is also available for farmers who do not own a smartphone. The SMS alert service sends text messages to farmers informing about the irrigation requirement (volume and time). Moreover, a web portal is available for information access. There are two interfaces for the portal: admin and user. The admin interface is used by the research team to view data for all the farmers, whereas the user interface is for the farmer. Therefore, the user interface of the agriculture DSS addresses the personalized requirements of each farmer.

## 3. Results and Discussion

During the 9-month study for the selected lemon orchard, the irrigation quantities applied as per the farmer estimates using the traditional method in Area A and as per the DSS algorithm in Area B were recorded. At the end of the season, lemon yield was also estimated in both farm areas to assess the impact of both irrigation methods.

### 3.1. Comparison of Irrigation Volume for Traditional and Agriculture DSS Methods

Table 1 shows the total irrigation volume used in both areas. As indicated, Area A, irrigated using the traditional farming method, utilized 96,569 L of irrigation water in the entire season. On the other hand, Area B, irrigated using the DSS algorithm, utilized only 44,290 L, which is 46% of the irrigation water used by the traditional method. In other terms, irrigation scheduled by the DSS algorithm saved 22,810,839 cubic feet of water. This establishes that irrigation scheduled using the DSS algorithm, based on real-time farm parameters, optimizes the use of available resources and saves water without affecting crop yield. In addition, it was observed that the crop yield was in fact increased, as discussed later.

**Figure 8:**
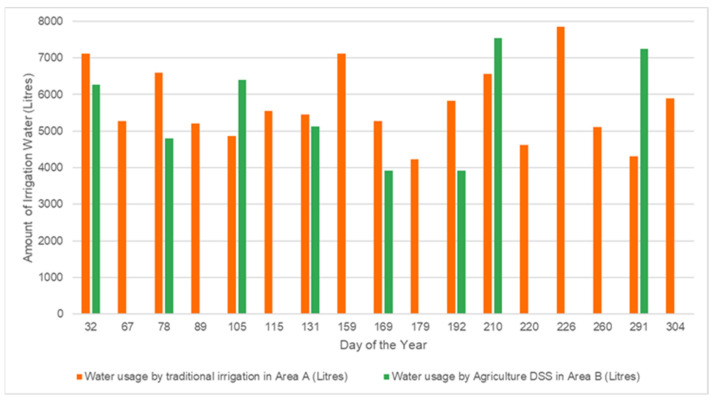
Water usage efficiency of traditional and DSS algorithm-based irrigation systems.

Table 2 lists the detailed irrigation schedule for both farms according to their application as per the day of the year and the water usage. Day 01 denotes 1 January of the year. As normal in the traditional irrigation method, the farmer irrigated the area irrespective of the actual requirement. On the other hand, since real-time sensor data were used to calculate water requirements in Area B, the DSS algorithm did not recommend irrigation when not needed. This data are also plotted in Figure 8 to illustrate the water usage efficiency.

### 3.2. Yield Increase

**Figure 9:**
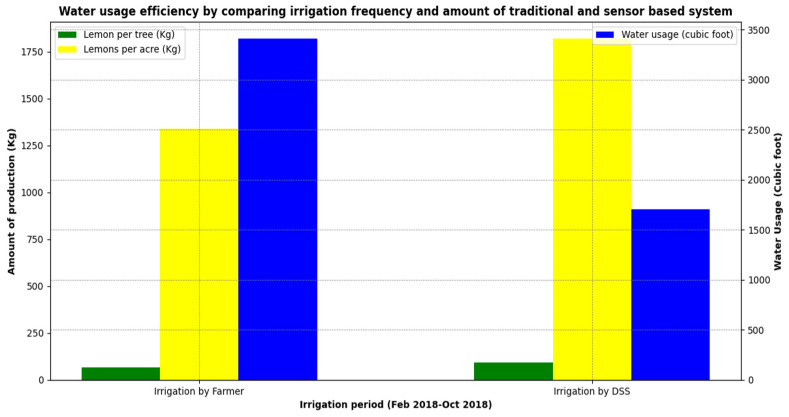
Yield increase for lemon crop.

Table 3 shows the resulting total yield at the end of the season in both farming areas (A and B). As is evident from the table, the lemon yield was much higher at 91 kg lemons per tree in Area B, which was irrigated as per recommendation by the DSS algorithm based on real-time parameters. In comparison, yield was at 67 kg per lemon tree in area A irrigated by traditional farming method. In short, the yield increased by 35% using DSS algorithm-based irrigation. The table further mentions that the total yield per acre in Area A as 4690 kg and that in Area B was 6370 kg. The total revenue generated in Area B (DSS algorithm-based irrigation) based on water saving and higher yield was 182,000 PKR, which was 35% higher than that in Area A (traditional irrigation). The farmers in Area B were delighted by the positive results of the IoT device and the associated DSS algorithm as they achieved 35% higher yield of lemons while consuming 50% less irrigation water. Figure 9 summarizes the results of crop yield and irrigation volume used in both the farm areas.

### 3.3. Future Prospects

The comparison study in Figure 9 shows the difference in irrigation by traditional farming and agriculture DSS; accordingly, in the future, these results could help in increasing the annual production of lemons all around the country. The area of cultivation for the citrus fruits in total is around 185,400 hectares (458,133 acres) in Pakistan. Moreover, 97.16% of the citrus fruits are cultivated in the province of Punjab, occupying an area of 183,200 hectares (452,697 acres) producing 2,097,700 tons annually [49]. Lemon represents about 1.2% of the total citrus fruit production in the Punjab, occupying an area of 4600 hectares (11,366.85 acres) and producing 2600 tons annually. Overall, the total area given over to lemon production in Pakistan is approximately 5000 hectares (12,355 acres) on 70 trees per acre. As indicated in our results, the traditional irrigation method typically generates 67 kg of lemon per tree; thus, 70 trees per acre could produce 4690 kg of lemon/acre. Table 4 shows a summary of potential savings according to the results for our trial. Although based on a single trial, the savings are significant and further savings could even be made if the technique is extended to other citrus crops.

Specifically, for this trial, the agriculture DSS significantly improved the yield of each tree, giving an improvement of 35%, notably achieving this by using 50% less water. In many ways, this water saving is the most important point because it may allow more traditionally unsuitable land to be exploited for production, without increasing the overall water usage.

## 4. Conclusions

Excessive irrigation is a major agricultural problem. It reduces the quality of some crops and reduces the crop yield, while wasting a scarce resource. Farmers in developing countries often irrigate excessively due to their ignorance of the problems of water logging and salinity. In most cases, with the exception of water-intensive crops, crops need only an adequate amount of water to grow. Additional water destroys the root growth and decreases the water security of a region. To address this, an IoT device was developed and paired with a DSS for irrigation scheduling of a lemon orchard in Gadap, Karachi. The agriculture DSS scheduled irrigation according to data collected from the sensors embedded in the device; soil and crop data are pre-fed to the device, whereas weather data are collected real time. The Penman–Monteith equation and crop coefficient approach are an essential part of the system and form the basis of a prediction system. Furthermore, the system was adjusted according to the Warabandi system practised in Sindh, Pakistan. The final results in the form of a comparative study showed that the DSS was successful. It saved 52,280 L of water on the farm while boosting the harvest by producing 1680 kg more lemon per acre, i.e., 35% greater turnout while preserving 50% irrigation water. Therefore, it is an effective system that increases water productivity for the tested crop; with slight adjustments, it could be simply applied to other non-water-intensive crops. As mentioned earlier, the developed product was launched in a start-up company as a service and is being adopted by many farmers in the country for crop monitoring and irrigation scheduling, providing evidence of the need for such a system. We intend to report further results as the system gains usage in more situations.

## Figures and Tables

**Table 1:** Irrigation statistics (L).

Traditional Irrigation (Area A)	DSS Scheduled Irrigation (Area B)	Water Saved	Percentage of Water Saved
96,569	44,290	52,279	46%

**Table 2:** Water usage efficiency by comparing irrigation frequency and amount of traditional and sensor-based system.

Day of the Year	Water Usage by Traditional Irrigation in Area A (L)	Water Usage by Agriculture DSS in Area B (L)
32	7110	6266
67	5273	0
78	6588	4799
89	5212	0
105	4873	6399
115	5553	0
131	5452	5120
159	7117	0
169	5277	3922
179	4224	0
192	5833	3924
210	6563	7546
220	4624	0
226	7856	0
260	5104	0
291	4316	7242
304	5894	0

**Table 3:** Yield increase for lemon crop.

Methods	Yield of Lemons per Tree	Yield of Lemons per Acre	Water Usage (L)	Water Usage (Cubic Feet)	Revenue (PKR)
**Traditional Farming**	67 kg	4690 kg	96,570	3410	134,000
**Irrigation by DSS**	91 kg	6370 kg	44,290	1705	182,000

**Table 4:** Yield increase for annual cultivation of lemon crop in Pakistan.

	Traditional Irrigation by Farmer (Area A)	Irrigation by DSS (Area B)	Difference
**Yield of Lemon per tree**	67 kg	91 kg	24 kg
**Total Yield (kg)**	4690 kg × 12,355.27 acre = 57,946,216.3 kg	6370 kg × 12,355.27 acre = 78,703,069.9 kg	20,756,853.9 kg
**Total Yield (tons)**	57,946.2 tons	78,703.1 tons	20,756.8 tons
**Total Water Usage (L)**	96,569.8 L × 12,355.27 acre = 1,193,145,952.846 L	44,290 L ×12,355.27 acre = 547,214,908.3 L	645,931,044.546 L
**Total Water usage (Cubic feet)**	42,135,551.7 ft^3^	19,324,712.1 ft^3^	22,810,839.6 ft^3^
**Revenue (PKR)**	5794 M	7870 M	2076 M
**Revenue (USD)**	37.95 M	51.54 M	13.59 M
